# MiR-486 and miR-92a Identified in Circulating HDL Discriminate between Stable and Vulnerable Coronary Artery Disease Patients

**DOI:** 10.1371/journal.pone.0140958

**Published:** 2015-10-20

**Authors:** Loredan S. Niculescu, Natalia Simionescu, Gabriela M. Sanda, Mihaela G. Carnuta, Camelia S. Stancu, Andreea C. Popescu, Mihaela R. Popescu, Adelina Vlad, Doina R. Dimulescu, Maya Simionescu, Anca V. Sima

**Affiliations:** 1 Lipidomics Department, Institute of Cellular Biology and Pathology “Nicolae Simionescu” of the Romanian Academy, Bucharest, Romania; 2 Centre of Advanced Research in Bionanoconjugates and Biopolymers, “Petru Poni” Institute of Macromolecular Chemistry, Iasi, Romania; 3 Cardiology Clinic, Elias University Emergency Hospital, Bucharest, Romania; National University of Singapore, SINGAPORE

## Abstract

Small non-coding microRNAs (miRNAs) are implicated in gene regulation, including those involved in coronary artery disease (CAD). Our aim was to identify whether specific serum miRNAs present in the circulating lipoproteins (Lp) are associated with stable or vulnerable CAD patients. A cardiovascular disease-focused screening array was used to assess miRNAs distribution in sera collected from 95 CAD patients: 30 with stable angina (SA), 39 with unstable angina (UA), 26 at one month after myocardial infarction (MI) and 16 healthy control subjects. We found that miR-486, miR-92a and miR-122 presented the highest expression in CAD sera. These miRNA together with miR-125a, miR-146a and miR-33a were further individually analyzed by TaqMan assays. The results were consistent with PCR-array screening data that all of these miRNAs were significantly increased in CAD patients compared to controls. Using a binary logistic regression model, we established that miR-486 and miR-92a in association with some high-density lipoprotein (HDL) components can designate vulnerable CAD patients. Further, all classes of Lp were isolated from sera by density gradient ultracentrifugation. Analysis of the selected miRNAs in each Lp class showed that they were associated mainly with HDL, miR-486 and miR-92a having the highest levels. In UA and MI patients, miR-486 prevailed in HDL_2_, while miR-92a prevailed in HDL_3_, and their levels discriminate between stable and vulnerable CAD patients. We identified two circulating miRNAs that in association with some lipid metabolism biomarkers can be used as an additional tool to designate vulnerable CAD patients.

## Introduction

Atherosclerosis is an inflammatory disease, generated and/or aggravated by lipid metabolism disorders [1, 2]. Coronary artery disease (CAD) is the result of atherosclerotic plaque development in the wall of coronary arteries. Because CAD progression is highly variable, non-invasive approaches to identify individuals with unstable plaques and therefore, at risk of acute coronary syndromes (ACS), are important. The discovery of microRNAs (miRNAs) and their role in the regulation of gene expression is one of the most exciting scientific breakthroughs of the last two decades. MiRNAs are small (18–25 nucleotides), single-stranded, non-coding RNAs that post-transcriptionally regulate gene expression by inhibiting translation and/or by promoting mRNA degradation through partial or complete pairing with the 3’-UTR of mRNA [3, 4]. Currently, there are over 2,500 mature human miRNA sequences listed in the miRNA registry (Sanger miRBase release 21, www.mirbase.org). MiRNAs have been shown to circulate in a highly stable, cell-free form in many body fluids, including the blood [5], usually associated with microparticles, exosomes, lipoproteins (Lp) or protein complexes [6, 7].

Specific profiles of circulating miRNAs have been associated with several diseases, such as cancer, neurodegenerative disorders, atherosclerosis and diabetes [8–10]. Published reports point to the role of miRNAs in the development of cardiovascular diseases (CVD) and their use as suitable tools for accurate diagnosis [11]. Only very few studies have investigated the association of miRNAs with circulating Lp and their implication in CAD evolution [12–14].

In this study, we aimed to identify circulating miRNAs that are associated with specific classes of Lp isolated from the sera of 3 groups of patients in various stages of CAD: patients with stable (SA) or unstable angina (UA) and patients at one month after myocardial infarction (MI), compared to healthy control subjects. An additional aim was to determine specific miRNAs that together with a group of known lipid metabolism biomarkers are able to predict the risk for vulnerable CAD. To this purpose, intermediate- (IDL), low- (LDL) and high density lipoproteins (HDL) were isolated from CAD patients’ sera and the presence of miRNAs and their distribution in the Lp classes was investigated.

## Material and Methods

### Study design and subjects

The investigation included 111 subjects (38 women and 73 men, aged 24–79 years): 95 patients with CAD (30 SA, 39 UA and 26 MI) and 16 healthy control subjects. SA group was considered as reference category and UA and MI groups together as vulnerable CAD groups (risk category). We did not study patients with acute myocardial infarction (AMI) due to many limitations: the precise time point of the blood collection, the perfusion of heparin during the acute phase of the myocardial infarction (which re-circulated up to hours or even days after the event) that disturbs the serum miRNAs profile of patients, as reported by Kaudewitz et al. [15]. Therefore, we recruited only patients at one month after the acute myocardial infarction (MI group). The MI patients were considered as positive control for the vulnerable category of patients, as they already experienced an acute myocardial infarction one month before their enrollment in this study. All CAD patients were from the Cardiology Clinic, Elias Emergency University Hospital, Bucharest. As control we used healthy donors (aged 24–62 years, 13 women and 3 men) from Blood Transfusion Center, Bucharest with no CVD risk factors or other documented disorders. General exclusion criteria were autoimmune or malignant diseases, acute infections and severe hepatic or renal diseases.

From each subject, fasting blood samples were collected and serum was isolated for biochemical and miRNAs analysis. None of the patients received heparin or fractionated heparin at the time of sampling. For Lp isolation and serum miRNAs profiling, equal amounts of sera from subjects of each group were pooled; the procedure was performed on three independent pools of sera from each group.

This study was carried out in accordance with the principles from the Declaration of Helsinki (The Code of Ethics of the World Medical Association, last updated at the 64^th^ WMA General Assembly, Fortaleza, Brazil, October 2013) for experiments involving humans. All participants gave their written informed consent by signing the appropriate paperwork and respecting their anonymity and privacy rights. The Ethics Committees of the Institute of Cellular Biology and Pathology “N. Simionescu” and of the Elias University Emergency Hospital have approved the study. Detailed information about the study design and subjects is provided in the S1 File.

### Determination of serum parameters

Concentrations of total cholesterol (TC), HDL cholesterol (HDL-C), LDL cholesterol (LDL-C), triglycerides (TG), non-esterified fatty acids (NEFA), fasting glucose, apolipoproteins (apoA-I, apoB and apoE), the cholesteryl ester transfer protein (CETP) activity were measured with commercial kits and the paraoxonase 1 (PON1) activity by an adapted method [16] in sera of all studied CAD patients and controls. Detailed assays are provided in the S1 File.

### Isolation and characterization of serum lipoproteins

Equal amounts of sera were pooled from subjects of each group and IDL, LDL and subpopulations of HDL, HDL_2_ and HDL_3_ were isolated by isopycnic density gradient ultracentrifugation as described [17]. Cholesterol and apolipoproteins levels and activity of CETP and PON1 were determined in the isolated Lp classes. Alternatively, Lp were isolated by a sequential flotation ultracentrifugation from control sera [18]. Detailed methods are provided in the S1 File.

### Analysis of miRNAs in sera and lipoproteins

The miRNAs were isolated from pooled or individual sera and from selected Lp fractions using miRNeasy Serum/Plasma kit (Qiagen, Dusseldorf, Germany), according to the manufacturer’s instructions. As a spike-in to correct sample-to-sample variation, 25 fmol of synthetic *cel-miR-39* (Life Technologies, Carlsbad, CA, USA) was exogenously added to each sample during miRNAs purification, as previously described [14, 19–21]. Screening of serum miRNAs distribution was done on pooled sera from 8 randomly selected subjects per group using the *Pathway-focused Human CVD miScript miRNA PCR array* (Qiagen, Dusseldorf, Germany) following the manufacturer’s protocol without pre-amplification. Relative expression of a specific human miRNA was given proportionally to that of exogenously added *cel-miR-39*. Fold change values of individual miRNA from the pooled sera of CAD patients were expressed relatively to those from pooled sera of control group. TaqMan miRNA Assays (Life Technologies) were used to assess the individual levels of *Homo sapiens* (hsa)-miR-486-5p, hsa-miR-92a-3p, hsa-miR-122-5p, hsa-miR-125a-5p, hsa-miR-146a-5p, hsa-miR-33a-5p and cel-miR-39-5p, in all 111 study subjects (95 CAD patients and 16 control subjects), according to the manufacturer’s instructions. The expression level of each individual miRNA was determined relative to that of exogenously added cel-miR-39 and calculated by using the 2^-ΔCq^ method [22], then log-transformed for the statistical analysis. The detailed methods are provided in the S1 File.

### Statistical analysis

Statistical analysis was done using the statistical software SPSS for Windows v21.0 (IBM SPSS, IBM Ireland, Dublin, Ireland). The continuous distributed quantitative variables (biochemical and miRNAs data) were expressed as means ± standard error of the mean (SEM) and analyzed by Oneway ANOVA with *Least Significant Difference* (LSD) Post-hoc test. Crosstabs distribution with chi-squared (χ^2^) analysis was performed to evaluate the differences between logistic data (gender, age distribution, presence of obesity, diabetes or hypertension, use of medication). The values obtained for circulating miRNAs levels in all subjects’ sera were log-transformed. Parametric bivariate correlation analysis of log-transformed miRNA levels with serum lipid parameters was performed using the Pearson’s function and corresponding p-value. To analyze the potential of circulating miRNAs to designate vulnerable CAD patients, we employed a binary logistic regression model (LR) with the enter iteration method, considering SA group as reference category, and UA and MI groups together as risk (vulnerable) category, with serum miRNAs, lipids, apolipoproteins and Lp-associated enzymes activity introduced in the LR model as covariates. The threshold for statistical significance was set to 5% (p-values lower than 0.05).

## Results

### MiRNAs profiling in CAD patients’ sera

**Fig 1:**
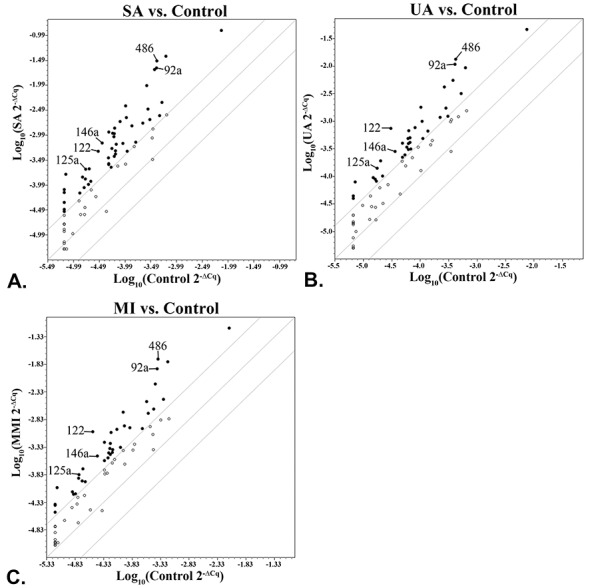
Distribution of circulating miRNAs profiled in the pooled sera from coronary artery disease (CAD) patients: with stable angina (SA) (A), unstable angina (UA) (B), and patients at one month after myocardial infarction (MI) (C), illustrated as scatter plots of the fold regulation values expressed relative to control group (boundary = 4). The miRNAs selected for individual validation are depicted by arrows. Black dots = up-regulated miRNA (fold regulation over 4), empty dots = unchanged miRNA (fold regulation between -4 and 4, between the boundaries).

The clinical data and the serum lipids, apolipoproteins and HDL-associated enzymes of the 111 subjects under study are presented in Tables 1 and 2. All CAD patients were significantly older and overweight compared to control subjects (p<0.05, Table 1). The results of the circulating miRNAs profiles in the sera of CAD patients are illustrated as scatter-plots in Fig 1, as hierarchical heatmap in Fig A in S1 File and shown in detail in Table A in S1 File. We found a high number of up-regulated miRNAs (fold change > 4 relative to control subjects), namely 48 in SA, 38 in UA and 38 in MI (Fig 1A–1D, Table A in S1 File). Top ranked miRNAs were considered those from the MI group. Out of all the up-regulated miRNAs, the highest ranked (miR-486, miR-92a and miR-122) were selected for analysis in all subjects. In addition, miR-146a and miR-125a, ranked 14^th^ and 16^th^, respectively, and miR-33a were selected based on published data in CAD patients [9, 12, 23, 24]. Data from the CVD-dedicated PCR array showed that there were no significantly down-regulated miRNAs in all CAD patients’ sera (fold change < 0.5 relative to control subjects; Fig 1, Table A in S1 File).

**Table 1:** Clinical characteristics of the CAD patients and control subjects.

Parameter	Control (n = 16)	SA (n = 30)	UA (n = 39)	MI (n = 26)
**Age** (years)	**43.31** ± 2.79	**62.23** ^***^ ± 1.48	**62.49** ^***^ ± 1.56	**57.31** ^***^ ^§^ ± 2.33
**Gender Male** (N, %)	**3** (18.8%)	**20** (66.7%)	**28** (71.8%)	**22** (84.6%)
**BMI**(kg/m^2^)	**24.09** ± 0.90	**29.49** ^*^ ± 1.01	**28.34** ^*^ ± 0.83	**31.39** ^**^ ^§^ ± 1.51
**Overweight** (25<BMI<30 kg/m^2^) (N, %)	**2** (12.5%)	**8** (28.6%)	**11** (28.2%)	**6** (23.1%)
**Obese** (BMI>30 kg/m^2^) (N, %)	**0** (0%)	**13** (43.3%)	**17** (43.6%)	**13** (50.0%)
**Glucose** (mmol/L)	**5.305** ± 0.142	**6.280** ± 0.363	**6.396** ± 0.429	**6.744** ± 0.640
**Diabetes mellitus** (N, %)	**0** (0%)	**12** (40.0%)	**10** (25.6%)	**12** (46.2%)
**Hypertension** (N, %)	**0** (0%)	**26** (86.7%)	**28** (71.8%)	**14** (53.8%)
**LVEF** (%)	**NA**	**55.83** ± 1.40	**53.10** ± 1.64	**53.61** ± 2.23
**Troponin I** (μg/L)	**ND** (< 0.200)	**0.283** ± 0.082	**0.287** ± 0.029	**0.710** ^§^ ± 0.360
**Statin therapy** (N, %)	**0** (0%)	**23** (76.7%)	**26** (66.7%)	**22** (84.6%)
**Aspirin** (N, %)	**0** (0%)	**19** (63.3%)	**24** (61.5%)	**21** (80.7%)
**Anti-platelets therapy** (N, %)	**0** (0%)	**13** (43.3%)	**21** (53.8%)	**21** (80.7%)
**ACE-Inhibitor** (N, %)	**0** (0%)	**24** (80.0%)	**26** (66.7%)	**18** (69.2%)

SA = patients with stable angina, UA = patients with unstable angina, MI = patients at 1 month after myocardial infarction, NA = not available, ND = not detectable. Data are expressed as means ± standard error of the mean (SEM) and analyzed with Oneway ANOVA test with LSD Posthoc analysis. Chi-squared (χ^2^) analysis was performed to evaluate the differences between logistic data (gender, age distribution, obesity, diabetes, hypertension, medication).

^*^ p<0.05

^**^ p<0.01

^***^ p<0.001 vs. Control

^**§**^ p<0.05 vs. UA

**Table 2:** Lipids, apolipoproteins and activity of Lp-associated enzymes in sera collected from CAD patients and control subjects.

Parameter	Control (n = 16)	SA (n = 30)	UA (n = 38)	MI (n = 25)
**TC** (mmol/L)	**5.035** ± 0.212	**3.942** ^**^ ± 0.165	**4.802** ^‡‡^ ± 0.163	**4.057** ^**^ ^§^ ± 0.326
**LDL-C** (mmol/L)	**3.202** ± 0.166	**2.057** ^***^ ± 0.126	**2.972** ^‡‡^ ± 0.179	**2.146** ^**^ ^§§^ ± 0.287
**HDL-C** (mmol/L)	**1.405** ± 0.095	**1.155** ^*^ ± 0.065	**1.103** ^**^ ± 0.067	**1.009** ^**^ ± 0.077
**TG** (mmol/L)	**0.937** ± 0.110	**1.557** ^*^ ± 0.168	**1.879** ^**^ ± 0.150	**1.704** ^**^ ± 0.211
**PL** (g/L)	**1.794** ± 0.116	**1.768** ± 0.119	**1.725** ± 0.126	**1.723** ± 0.124
**NEFA** (mmol/L)	**0.342** ± 0.038	**0.281** ± 0.026	**0.289** ± 0.031	**0.288** ± 0.027
**ApoA-I** (g/L)	**1.170** ± 0.086	**1.103** ± 0.060	**1.148** ± 0.064	**0.844** ^**^ ^‡‡^ ^§§^ ± 0.058
**ApoB-100** (g/L)	**0.891** ± 0.043	**1.017** ± 0.057	**1.197** ^**^ ^‡^ ± 0.059	**0.880** ^§§§^ ± 0.049
**ApoE** (g/L)	**0.015** ± 0.002	**0.026** ^*^ ± 0.003	**0.034** ^***^ ^‡^ ± 0.004	**0.020** ^§§§^ ± 0.002
**CETP activity** (μmol/L/h)	**73.64** ± 4.40	**63.79** ± 3.29	**70.14** ± 3.24	**60.36** ± 3.32
**PON1 activity** (U/L)	**739.39** ± 126.27	**597.11** ^*^ ± 76.04	**560.83** ^*^ ± 50.64	**457.38** ^*^ ± 48.66

SA = patients with stable angina, UA = patients with unstable angina, MI = patients at 1 month after myocardial infarction. Data are expressed as means ± standard error of the mean (SEM) and analyzed with Oneway ANOVA test with LSD Posthoc analysis.

^*^ p<0.05

^**^ p<0.01

^***^ p<0.001 vs. Control

^**‡**^ p<0.05

^**‡‡**^ p<0.01 vs. SA

^**§**^ p<0.05

^**§§**^ p<0.01

^**§§§**^ p<0.001 vs. UA

### Selected miRNAs levels in CAD patients’ sera

**Fig 2:**
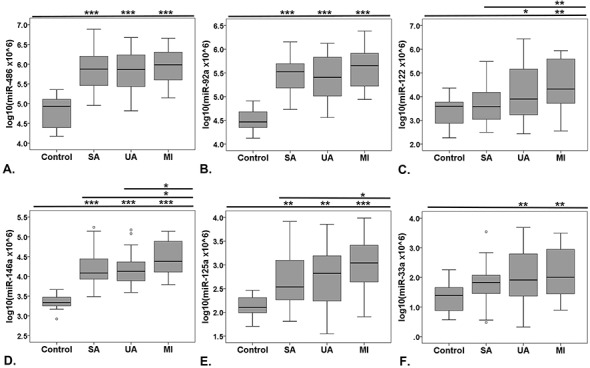
Boxplot distribution of the highest ranked miRNAs after miRNAs screening analysis. Levels of miR-486 (**A**), miR-92a (**B**), miR-122 (**C**), miR-125a (**D**), miR-146a (**E**) and miR-33a (**F**) measured in the sera from control subjects and coronary artery disease (CAD) patients with stable angina (SA), unstable angina (UA) and patients at one month after myocardial infarction (MI). Data are expressed as log-transformed individual 2^-ΔCq^ values. Note that significantly increased serum miRNAs levels were measured in all CAD patients vs control sera, but they do not differ statistically between the CAD groups (*p<0.05, **p<0.01, ***p<0.001 vs. control group, Oneway ANOVA analysis with LSD Posthoc test).

The data resulting from selected miRNA TaqMan assays were expressed as log-transformed values of multiplied 2^-ΔCq^ values (to obtain a distribution of positive values). The analysis of serum miRNAs (presented as boxplots in Fig 2A–2F) revealed that the levels of all selected miRNAs were significantly higher in the sera of all CAD patients compared to the controls. However, no statistically significant differences of the mean miRNAs levels were detected between the stable and the vulnerable CAD patients, particularly between SA and UA patients (Fig 2A–2F). We observed a small, but significant, increase of miR-122, miR-146a and miR-125a levels in sera from MI vs. SA group (p<0.05) (Fig 2C–2E).

The bivariate parametric correlation analysis between log-transformed miRNA levels and lipid metabolism biomarkers showed that serum PON1 activity correlated negatively with miR-486 (r = -0.244, p = 0.014), miR-92a (r = -0.269, p = 0.007) and miR-122 (r = -0.275, p = 0.005), while apoA-I levels correlated negatively with miR-146a (r = -0.252, p = 0.010). Also, miR-122 correlated with apoE (r = -0.246, p = 0.011) and CETP activity (r = 0.291, p = 0.012). We analyzed the correlation between the selected miRNAs and troponin I levels in the patients’ sera, but no significant correlation was found, probably because all vulnerable CAD patients were enrolled one month after AMI and during this period received a clinically successful standard treatment, so that troponin I decreased to almost basal levels.

The binary LR model used to analyze miRNAs levels in the studied cohort showed that miR-486 and miR-92a had the highest individual contribution to the designation of vulnerable CAD patients and was expressed by the exponent values of the beta coefficients (expB or odd ratio: 4.25 for miR-486 and 2.38 for miR-92a), in good agreement with the data obtained from the miRNAs screening (Table A in S1 File). However, the LR model including only the miRNAs with no other covariates did not provide a statistically significant discrimination between the vulnerable and stable CAD groups (accuracy 70.5%, p = 0.270). The LR model including miR-92a and miR-486 serum levels with adjustment for age, gender, together with some serum lipids and apolipoproteins levels (HDL-C/LDL-C, apoA-I, apoE) and HDL-associated enzyme activity (PON1) as covariates resulted in a significant designation of vulnerable CAD patients with an accuracy of 84% (p = 0.010). Any other adjustments for obesity, diabetes mellitus, hypertension or medical therapies did not alter the resulting data of the LR model. No control subjects were considered in this type of analysis (LR model). Noteworthy, this estimate for vulnerable CAD patients was statistically significant only when miR-92a and miR-486 were taken together as covariates in the devised LR model. Moreover, the addition of miR-122 (step p = 0.007) improved the LR estimate for vulnerable CAD patients (accuracy 86%, p = 0.005).

### MiRNAs distribution in HDL subfractions from CAD patients’ sera

**Fig 3:**
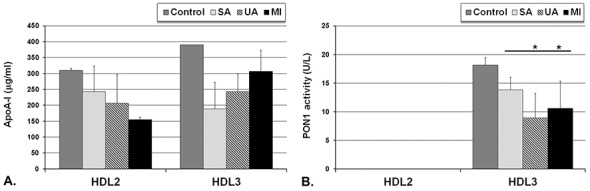
Levels of apolipoprotein A-I (apoA-I) (A) and paraoxonase-1 (PON1) activity (B) in HDL_2_ and HDL_3_ fractions isolated by isopycnic density gradient ultracentrifugation from pooled sera of control subjects and the coronary artery disease (CAD) patients with stable angina (SA), unstable angina (UA) and patients at one month after myocardial infarction (MI); the procedure was performed on 3 independent pools of sera from each group. Data are expressed as means ± standard deviation and analyzed with Oneway ANOVA analysis with LSD Posthoc test; * p<0.05 vs. SA group.

Lp classes obtained after isopycnic density gradient ultracentrifugation of pooled sera from each group, namely IDL, LDL, HDL_2_ and HDL_3_ were collected and further analyzed for miRNAs content. We determined lower apoA-I levels in HDL_2_ from SA (41%), UA (54%) and MI (53%) compared to control group, and the same decrease happened in HDL_3_: in SA (58%), UA (52%) and MI (40%) (Fig 3A). PON1 activity was lower in HDL_3_ from SA (24%), UA (50%) and MI (42%) compared to control group (Fig 3B). PON1 activity in HDL_3_ isolated from UA and MI was lower by 50% and 40%, respectively, compared to SA. PON1 activity differed significantly between the two HDL subpopulations, being detected only in HDL_3_ and absent in HDL_2_ from all sera (Fig 3).

**Fig 4:**
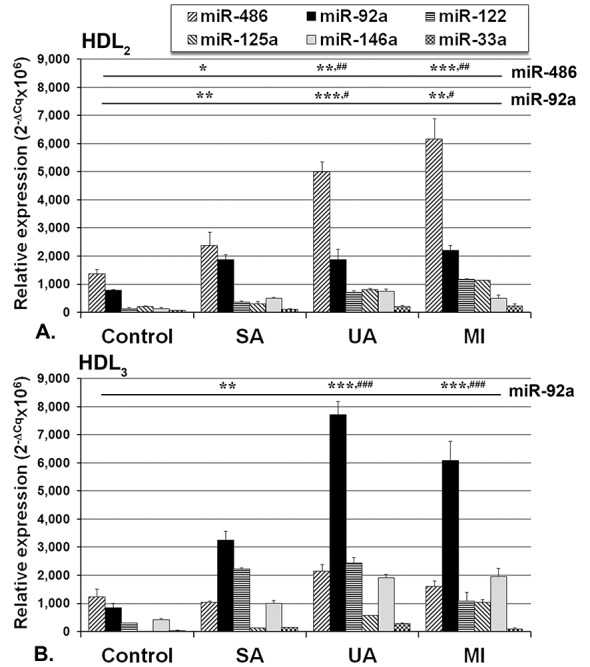
Distribution of selected miRNAs in HDL populations: HDL_2_ (A) and HDL_3_ (B) isolated by density gradient ultracentrifugation from pooled sera obtained from coronary artery disease (CAD) patients with stable angina (SA), unstable angina (UA) and patients at one month after myocardial infarction (MI) and control; the procedure was performed on 3 pools of sera from each group. Data are expressed as mean 2^-ΔCq^ values multiplied with the same coefficient as for serum (*p<0.05, **p<0.01, ***p<0.001 vs. control group; ^##^p<0.01, ^###^p<0.001 vs. SA group, Oneway ANOVA test with LSD Posthoc analysis).

To validate the determined miRNAs distribution in Lp isolated by isopycnic ultracentrifugation, we performed a sequential flotation ultracentrifugation for control sera. We found a similar distribution of the selected miRNAs in IDL, LDL, HDL_2_ and HDL_3_ isolated with either of the two methods (data not shown). Analysis of the distribution of miRNAs in the isolated Lp revealed that all selected miRNAs were mainly associated with HDL (Fig 4), while their levels in IDL and LDL were ten times lower (Table B in S1 File). The most abundant miRNA in IDL and LDL was miR-92a.

The distribution of the selected miRNAs differed between HDL subfractions, HDL_2_ and HDL_3_ (Fig 3A and 3B). In HDL from all CAD patients miR-486 and miR-92a had the highest levels, with miR-486 prevailing in HDL_2_ and miR-92a in HDL_3_ (Fig 4A and 4B).

In HDL_2_, miR-486 had the highest level compared to controls in MI (5x, p = 3.72x10^-4^), followed by UA (3.6x, p = 1.05x10^-3^) and SA (1.8x, p = 0.0112). Moreover, when compared to SA group, in vulnerable groups’ sera the level of miR-486 in HDL_2_ was significantly higher: UA (2.1x above SA, p = 4.90x10^-3^) and MI (2.6x above SA, p = 1.77x10^-3^) (Fig 4A). The second most abundant miRNA in HDL_2_ was miR-92a, that was increased in all CAD groups (2–2.8x above control, p<0.05 for all CAD groups) (Fig 4A).

In HDL_3_, miR-92a had higher levels compared to control in UA (10x, p = 8.75x10^-4^), MI (7.5x, p = 4.49x10^-4^) and SA (3.8x, p = 7.08x10^-3^). MiR-122 was ranked second in UA (8x, p = 0.0104) and SA (7.5x, p = 5.62x10^-3^), and miR-146a was ranked third, almost equally abundant in UA and MI (4.7x, p = 5.58 x10^-3^, respectively 0.0179) (Fig 4B). In HDL_3_, miR-486 levels were lower than in HDL_2_ in all CAD groups [MI (3.9x), UA (2.3x) and SA (2.3x)] (Fig 4B). When compared to SA group, the level of miR-92a in HDL_3_ was significantly higher in UA (2.4x above SA, p = 7.27x10^-4^) and MI (1.9x above SA, p = 4.21x10^-3^) (Fig 4B). The distribution of miR-92a and miR-486 in HDL_3_ from control subjects was reversed as compared to the patients’ one, miR-486 being predominant (Fig 4B).

## Discussion

Several studies have suggested the potential of miRNAs from blood to be used as biomarkers for CAD [25], but only few succeeded in finding correlations between circulating miRNAs and other serum parameters already accepted as biomarkers for CAD [12]. Data obtained in the present study show that: (i) miR-486, miR-92a, miR-122 have the highest expression in CAD patients’ relative to control sera; (ii) the mean levels of each selected miRNA do not differ statistically between the groups of CAD patients; (iii) taken together, miR-486, miR-92a levels and HDL-C, apolipoproteins and PON1 activity could designate vulnerable as opposed to stable CAD patients; (iv) miR-486, miR-92a, miR-122, miR-125a and miR-146a are associated mainly with serum HDL and ten times less with IDL and LDL; (v) miR-486 and miR-92a have the highest levels in HDL isolated from vulnerable (UA and MI) compared to SA patients and their distribution differs between HDL subfractions from the respective groups, miR-486 prevailing in HDL_2_, while miR-92a prevails in HDL_3_.

A limitation of our study, as well as in many others, is the relatively small number of patients evaluated and, therefore, it should be considered as a pilot study to be validated in a larger cohort of patients. Nevertheless, it is notable that although the number of subjects was rather small, the employed LR model and the distribution of miR-486 and miR-92a in HDL gave statistically relevant data. We investigated the contribution of obesity, diabetes mellitus, hypertension or medication, considering them as co-factors in the LR statistical model. The results show that the adjustments for BMI, diabetes mellitus, hypertension or medication did not alter the two miRNAs contribution in the LR model. Another drawback of miRNAs studies in general is that, despite the many techniques used, there are no standardized methods for the quantification of circulating miRNAs, making the comparison between the results from different laboratories difficult.

To date, only few data exist regarding circulating miR-486 levels in CAD. Lai et al. reported a distinct serum miRNA expression signature in adults undergoing cardiac bypass surgery, serum miR-486 being negatively correlated with systemic ventricular contractility [26]. Circulating members of miR-17/92 cluster, and especially miR-92a, have been shown to be associated with CAD [20, 27]. In good agreement with our results, Ren et al. observed significantly elevated expression of circulating miR-17/92 cluster in plasma from UA compared to SA patients and healthy subjects [27]. In contrast to our results, Fichtlscherer et al. showed that circulating levels of miR-92a were significantly reduced in the CAD patients’ plasma compared with healthy subjects plasma [20]. Other published data suggest that miR-92a has a pro-atherosclerotic function and is associated with the endothelial dysfunction [28]. A large-scale miRNA profiling in human umbilical vein endothelial cells identified miR-92a as an atherosclerosis-associated miRNA (athero-miR) candidate, whose expression is preferentially upregulated by the combination of low shear stress and oxidized LDL [29]. We observed that oxidized LDL levels were increased (unpublished observations) and PON1 activity decreased in CAD patients’ sera, especially in the vulnerable groups. PON1 decreased activity found in plasma of atherosclerotic patients compared to normal subjects has been associated with the presence of dysfunctional HDL [30] and we report that serum PON1 activity correlates negatively with miR-92a, miR-486 and miR-122 levels. Moreover, our data from the serum miRNAs profiling indicated that miR-122, a dominant hepatocyte-specific miRNA [31], is the third abundant miRNA in the UA patients’ sera. We also found that miR-122 was the second ranked miRNA in HDL_3_ isolated from UA patients’ sera. Also, results from our group, as well as from Gao et al., reported that increased plasma levels of miR-122 are associated with the presence of CAD in hyperlipidemic patients [24, 32].

We have also investigated the expression of miR-125a and miR-146a in the sera of CAD patients based on the reports showing that these miRNAs are related to cardiac injury or myocyte cell death [23]. Our results showed that serum levels of miR-146a and miR-125a were increased in CAD patients compared to control, and they were higher in MI compared to SA group, in agreement with Oerlemans et al., who reported that circulating levels of miR-146a in patients with acute coronary syndrome (ACS) are increased compared to non-ACS patients [33]. Bidzhekov et al. showed that miR-125a is overexpressed in human carotid artery plaque relative to internal mammary artery controls [34].

Our data revealing that all selected miRNAs are mainly associated with HDL are in good agreement and extend recent reports [6, 14]. To date, a single study reports the miRNAs distribution in lipoproteins by employing a microarray containing a larger number of miRNAs to investigate their association with HDL in familial hypercholesterolemia [6]. Vickers et al. showed that miR-122, miR-486, miR-92a, miR-125a-5p and miR-146a are associated with HDL and to a lesser extent with LDL isolated from normal and familial hypercholesterolemic subjects, while miR-33a was undetectable [6]. In contrast, we detected miR-33a both in HDL and LDL from all sera. In another study, Wagner et al. analyzed 9 selected miRNAs (miR-92a, miR-126, miR-223, miR-150, miR-146a, miR-378, miR-145, miR-155, miR-30c) in healthy controls, SA and ACS patients and found that the miRNAs signatures varied only slightly between HDLs from patients’ sera [14]. Our experiments extend these data, identifying a preferential distribution of miRNAs in the HDL subpopulations (HDL_2_, HDL_3_), which proved to be a significant discriminator between the vulnerable and stable CAD groups. Thus, we show here that the increased levels of miR-486 in HDL_2_ and miR-92a in HDL_3_ were particularly associated with vulnerable CAD patients’ sera (UA and MI groups). The mechanism that enhances the miRNAs levels in HDL could involve the cellular transfer of lipids to HDL and/or the activity of the lipid transfer proteins that exchange lipids between apoB-containing Lp and HDL. It is accepted that HDL carries miRNAs, but it is not known how they are associated with HDL particles. Recent reports show that some HDL-associated miRNAs function as communication messengers over long distances from donor to recipient cells, but the authors could not indicate from what tissue they originate or if they are involved in the regulation of HDL secretion [6, 14, 35]. In a recent report, Tabet et al. showed that native HDL can transfer miR-223 to primary human coronary aortic endothelial cells and reduce inflammation by directly targeting intercellular adhesion molecule-1 [35]. Vickers et al. showed that HDL could transfer miRNAs to cultured hepatocytes, where they are functional and able to affect gene expression [6]. In contrast, Wagner et al. failed to demonstrate the efficient delivery of HDL-bound miRNAs to human umbilical vein endothelial cells [14]. It is not known whether miRNAs delivered to cells are of HDL-origin, because exosomes or microparticles could also transfer their miRNAs cargo to the same cells. Until now, there is no consensus in the literature whether miRNAs regulate HDL synthesis or HDL regulates cellular miRNAs expression. The miRNAs that regulate HDL metabolism are recently reviewed [36, 37]. There could be miRNAs that regulate genes involved in HDL’s apolipoproteins’ synthesis, but there are currently no data available. In the light of recent reports, the role of HDL-associated miRNAs in normal and pathological conditions deserves further studies.

In conclusion, we identified two circulating miRNAs, miR-486 and miR-92a, which together with apolipoproteins (apoA-I, apoE), PON1 activity and the ratio HDL/LDL cholesterol, can be used as an additional tool to designate vulnerable CAD patients. We also identified a preferential distribution of miR-486 and miR-92a in the HDL subpopulations (HDL_2_, HDL_3_) that can discriminate vulnerable versus stable CAD patients.

## Supporting Information

S1 File Supporting information file for the manuscript. This file contains Detailed methods with 11 References, Tables A and B and Fig A.
(DOC)
