# Supplementary material for: MiR-486 and miR-92a Identified in Circulating HDL Discriminate between Stable and Vulnerable Coronary Artery Disease Patients
Source: PLoS One. 2015 Oct 20;10(10):e0140958. doi: 10.1371/journal.pone.0140958 (PMC4617647; doi:10.1371/journal.pone.0140958)
Supplement: S1 File — This file contains Detailed methods with 11 References, Tables A and B and Fig A. (DOC) [file pone.0140958.s001.doc]

**S1 File - Supporting Information**

**MiR-486 and miR-92a identified in circulating HDL can discriminate between stable and vulnerable coronary artery disease patients**

Loredan S. Niculescu ^1 ¶^, Natalia Simionescu ^1,2 ¶^, Gabriela M. Sanda ^1^, Mihaela G. Carnuta ^1^, Camelia S. Stancu ^1^, Andreea C. Popescu ^3^, Mihaela R. Popescu ^3^, Adelina Vlad ^3^, Doina R. Dimulescu ^3^, Maya Simionescu ^1^, Anca V. Sima ^1 *^

^1^ Lipidomics Department, Institute of Cellular Biology and Pathology “Nicolae Simionescu” of the Romanian Academy, Bucharest, Romania

^2^ Centre of Advanced Research in Bionanoconjugates and Biopolymers, “Petru Poni” Institute of Macromolecular Chemistry, Iasi, Romania

^3^ Cardiology Clinic, Elias University Emergency Hospital, Bucharest, Romania

* Corresponding author

E-mail: [anca.sima@icbp.ro](mailto:anca.sima@icbp.ro)

^¶^ These authors contributed equally *to this work.*

**Detailed Methods**

**Study design and subjects**

The investigation included 111 subjects (38 women and 73 men, aged 24-79 years): 95 patients with CAD (30 SA, 39 UA and 26 MI) and 16 healthy control subjects. SA group was considered as reference category and UA and MI groups together as vulnerable CAD groups (risk category). The MI patients were considered as positive control for vulnerable category of patients, as they already experienced an acute myocardial infarction one month before their enrollment in this study. All CAD patients were from the Cardiology Clinic, Elias Emergency University Hospital, Bucharest. As control we used healthy donors (aged 24-62 years, 13 women and 3 men) from Blood Transfusion Center, Bucharest with no CVD risk factors or other documented disorders. General exclusion criteria were autoimmune or malignant diseases, acute infections and severe hepatic or renal diseases.

Clinical assessment, cardiac biomarkers (troponins) and ECG and echocardiography provided the criteria for inclusion in SA, UA and MI groups, according to the guidelines of the European Society of Cardiology. SA group patients presented typical angina, history of CAD and/or positive electrocardiography (ECG) stress test. UA patients had either a previous diagnosis of stable angina (that escalated in frequency, duration or severity of the episodes) or *de novo* angina (first episode less than two months back), but with no significant troponin elevation. Patients in MI group were diagnosed with acute myocardial infarction (AMI) as defined below and treated in the Cardiology Clinic, coming for follow up after 1 month. AMI was diagnosed as defined by the 3^rd^ universal definition: evidence of myocardial necrosis in a clinical setting consistent with acute myocardial ischemia. A detection of a rise and/or fall of cardiac biomarker values (preferably cardiac troponin) with at least one value above the 99^th^ percentile upper reference limit with at least one of the following: a) symptoms of ischemia, b) new or presumed new significant ST-segment–T wave changes or new left bundle branch block, development of pathological Q waves in the ECG, c) imaging evidence of new loss of viable myocardium or new regional wall motion abnormality.

From each subject, fasting blood samples were collected and serum was isolated for biochemical and miRNAs analysis. None of the patients received heparin or fractionated heparin at the time of sampling. For Lp isolation and serum miRNAs profiling, equal amounts of sera from patients of each group were pooled; the procedure was performed on three independent pools of sera from each group. Troponin I and left ventricle ejection fraction (LVEF) were measured in the hospital’s facilities.

This study was carried out in accordance with the principles from the Declaration of Helsinki (The Code of Ethics of the World Medical Association, last updated at the 64^th^ WMA General Assembly, Fortaleza, Brazil, October 2013) for experiments involving humans. All participants gave their informed consent, respecting their privacy rights. The Ethics Committees of the Institute of Cellular Biology and Pathology “N. Simionescu” and of the Elias University Emergency Hospital have approved the study.

**Determination of serum parameters**

In all CAD patients and control subjects, serum total cholesterol (TC), triglycerides (TG), and fasting glucose were measured employing automated biochemical analyzers, phospholipids (PL) and non-esterified fatty acids (NEFA) by end-point colorimetric method (Wako Chemicals GmbH, Neuss, Germany), HDL cholesterol (HDL-C) and LDL cholesterol (LDL-C) by commercially available kits (Dialab Gmbh., Neudorf, Austria), apolipoproteins A-I (apoA-I), apoB and apoE by commercial enzyme-linked immunosorbent assay (ELISA) kits (Mabtech AB, Nacka Strand, Sweden) and the cholesteryl ester transfer protein (CETP) activity by commercial fluorimetric assay kit (Biovision, Milpitas, CA, USA). The paraoxonase 1 (PON1) activity was determined by the capacity of the enzyme to hydrolyze the paraoxon substrate using an adapted method described by Rozenberg et al. .

**Isolation and characterization of serum lipoproteins**

We isolated and characterized intermediate- (IDL), low- (LDL) and high-density lipoproteins (HDL), with their subpopulations (HDL_2_ and HDL_3_) from the subjects’ group-pooled sera. An aliquot of 1.25 mL pooled sera from each Control, SA, UA and MI group was adjusted to a density of 1.23 g/mL with KBr (Sigma-Aldrich, St. Louis, MO, USA) and then overlaid with 2 mL of 1.21 g/mL KBr, 5 ml of 1.063 g/mL KBr, 1 mL of 1.019 g/mL KBr, and 1 mL of phosphate buffered saline (PBS). The mixture was ultracentrifuged for 18 h in a SW-41 Ti rotor at 30,000 rpm (154,000x g) in an Optima L-80XP ultracentrifuge (Beckman Coulter International SA, Nyon, Switzerland) . After that, 10 fractions (1 mL each) were collected from each tube and dialyzed against PBS pH 7.4, at 4°C in the dark. In addition, Lp from Control sera were isolated by sequential ultracentrifugation and then, miRNAs from the separated fractions were analyzed. The protein in the collected fractions was assessed by a modified Lowry method using bovine serum albumin as standard . Commercially available kits were used to measure cholesterol and apolipoprotein levels, CETP and PON1 activities were determined as described above. Lp were kept at -80°C until miRNAs analysis.

**Isolation of miRNAs from sera and lipoproteins**

The miRNAs were isolated from either 200 μL pooled sera, or 200 μL individual sera or from 300 μL Lp fractions (120 μg protein), using miRNeasy Serum/Plasma kit (Qiagen, Dusseldorf, Germany) according to the manufacturer’s instructions. To date, no reference miRNA is available and validated to normalize the miRNA content in biofluids, like serum or plasma. Therefore, for the normalization of sample-to-sample variation, 25 fmol of a synthetic *C. elegans* miRNA, cel-miR-39, mirVana miRNA mimic (Applied Biosystems, Life Technologies, Carlsbad, CA, USA) was exogenously added to each sample after the addition of Qiazol (Qiagen, Dusseldorf, Germany), as previously described . RNA was eluted from the silica columns with either 30 μL (for individual sera and Lp) or 20 μL (for pooled sera) of RNase-free water, and then stored at -80°C until analysis. RNA concentration and quality (optical densities at 260/280 nm) were assessed by a NanoDrop Lite spectrophotometer (Thermo Fisher Scientific Inc., Waltham, MA, USA).

**MiRNAs profiling in sera**

Equal amounts of sera were pooled from each group and miRNAs were isolated as described above. Screening of miRNAs was done using the *Pathway-focused Human Cardiovascular Disease miScript miRNA PCR array* (384-well format, MIHS-113ZE) and miScript SyBr Green PCR kit (both from Qiagen, Dusseldorf, Germany) following the manufacturer’s protocol without pre-amplification. Purified miRNA (5 μL) was reverse-transcribed using the miScript-II RT kit (Qiagen, Dusseldorf, Germany), according to the manufacturer’s instructions, and using a Veriti PCR system (Applied Biosystems, Life Technologies, Carlsbad, USA). The real-time PCR reaction was performed using the ViiA7 real-time PCR system (Applied Biosystems, Thermo Fisher Scientific, Waltham, MA, USA) and a PCR run setup according to the manufacturer’s instructions. Quantification cycle (Cq) values were determined after applying a fixed threshold level of 0.04 by means of the ViiA7 Software v1.2. Data were analyzed using the free data analysis software available online at the webpage: http://pcrdataanalysis.sabiosciences.com /mirna (Qiagen, Dusseldorf, Germany) and employing by the 2^-ΔCq^ calculation method . The level of miRNAs in SA, UA and MI sera were expressed relative to the level determined in control sera and to an exogenous control miRNA (*cel-miR-39*). All miRNAs with Cq values over 35 were excluded from further analysis. Fold change values of individual miRNA from the pooled sera of SA, UA and MI subjects were expressed relatively to those from Control sera. Fold change (2^-ΔΔCq^) values represent the normalized miRNA expression (2^-ΔCq^) in the patient sample divided to the normalized miRNA expression (2^-ΔCq^) in the C sample. Normalized expression of a specific human miRNA was given relative to that of exogenously added *cel-miR-39* (ΔCq = Cq^hsa-miRNA^ – Cq^cel-miR-39^). Fold change values greater than 2 indicated a positive or up-regulation, while fold change values less than 1 designated a negative or down-regulation of miRNA. Only significant fold change values (over 2 and below 0.5) were retained for further analysis of the miRNAs profiling, as suggested by the manufacturer. Two quality control tests were performed for the pooled sera used in the miRNA PCR array profiling. First, the reverse-transcription control (RTC) that gives an index of RT efficiency was expressed as ΔCq = average (Cq^RTC^) – average (Cq^PPC^), where PPC is the positive PCR controls; this index was lower than 7 (according to the manufacturer’s recommendations). A second quality test was performed for the degree of hemolysis in all pooled sera, being calculated as ΔCq = Cq^miR-23a^ - Cq^miR-451a^ and this was less than 7 for all sera .

**Individual miRNA reverse-transcription and real-time quantitative PCR**

Purified miRNAs isolated from individual sera or selected Lp were diluted 4:1 with RNase-free water. TaqMan miRNA assays and miRNA-specific stem-loop primers, TaqMan microRNA Reverse-Transcription Kit and Gene Expression Real-Time PCR Master Mix (all from Applied Biosystems, Thermo Fisher Scientific) were used to assess the levels of *Homo sapiens* (hsa)-miR-486-5p (ID001278), hsa-miR-92a-3p (ID000431), hsa-miR-122-5p (ID002245), hsa-miR-125a-5p (ID002198), hsa-miR-146a-5p (ID000468), hsa-miR-33a-5p (ID002135) and cel-miR-39-5p (ID000200), according to the manufacturer’s instructions. Reverse-transcription was done with miRNA-specific stem-loop primers of miRNA TaqMan assays on a Veriti PCR system (Applied Biosystems, Thermo Fisher Scientific). Real-time quantitative PCR was performed using the hydrolysis probes of miRNA TaqMan assays on a ViiA7 real-time PCR system (Applied Biosystems, Life Technologies) and for each sample, triplicate measurements were done on 96-well or 384-well reaction plates. The data were analyzed using the ViiA7 Software v1.2 (Applied Biosystems, Thermo Fisher Scientific) with the automatic Cq setting. The expression level of each individual miRNA was determined relative to cel-miR-39 and calculated using the 2^-ΔCq^ method (ΔCq = Cq^hsa-miRNA^ - Cq^cel-miR-39^) , then log-transformed for the statistical analysis.

**Statistical analysis**

Statistical analysis was done using the statistical software SPSS for Windows v21.0 (IBM SPSS, IBM Ireland, Dublin, Ireland). The continuous distributed quantitative variables (biochemical and miRNAs data) were expressed as means ± standard error of mean (SEM) and analyzed by two-tailed Oneway ANOVA with *Least Significant Difference* (LSD) Post-hoc test. Crosstabs distribution with Chi-squared (χ2) analysis was performed to evaluate the differences between logistic data (gender, age distribution, presence of obesity, diabetes or hypertension, use of medication). The values obtained for circulating miRNAs levels in all patients’ sera were log-transformed. Parametric bivariate correlation analysis of log-transformed miRNA levels with serum lipids parameters was performed using the Pearson’s function and corresponding p-value. To analyze the potential of circulating miRNAs to designate vulnerable CAD patients (estimated risk for vulnerable CAD), we employed a binary logistic regression model (LR) with the enter iteration method, considering SA group as reference category, and UA and MI groups together as risk (vulnerable) category, with serum miRNAs, lipids, apolipoproteins and Lp-associated enzymes activity introduced in the LR model as covariates. The threshold for statistical significance was set to 5% (p-values lower than 0.05).

**Supporting References**

1. Rozenberg O, Shih DM, Aviram M. Paraoxonase 1 (PON1) attenuates macrophage oxidative status: studies in PON1 transfected cells and in PON1 transgenic mice. Atherosclerosis. 2005;181(1):9-18. Epub 2005/06/09. doi: 10.1016/j.atherosclerosis.2004.12.030. PubMed PMID: 15939049.

2. Kypreos KE, van Dijk KW, Havekes LM, Zannis VI. Generation of a recombinant apolipoprotein E variant with improved biological functions: hydrophobic residues (LEU-261, TRP-264, PHE-265, LEU-268, VAL-269) of apoE can account for the apoE-induced hypertriglyceridemia. J Biol Chem. 2005;280(8):6276-84. Epub 2004/12/04. doi: 10.1074/jbc.M413458200. PubMed PMID: 15576362.

3. Havel RJ, Eder HA, Bragdon JH. The distribution and chemical composition of ultracentrifugally separated lipoproteins in human serum. J Clin Invest. 1955;34(9):1345-53. Epub 1955/09/01. doi: 10.1172/JCI103182. PubMed PMID: 13252080; PubMed Central PMCID: PMC438705.

4. Sima AV, Botez GM, Stancu CS, Manea A, Raicu M, Simionescu M. Effect of irreversibly glycated LDL in human vascular smooth muscle cells: lipid loading, oxidative and inflammatory stress. J Cell Mol Med. 2010;14(12):2790-802. Epub 2009/10/13. doi: 10.1111/j.1582-4934.2009.00933.x. PubMed PMID: 19818091; PubMed Central PMCID: PMC3822729.

5. Markwell MA, Haas SM, Bieber LL, Tolbert NE. A modification of the Lowry procedure to simplify protein determination in membrane and lipoprotein samples. Anal Biochem. 1978;87(1):206-10. Epub 1978/06/15. doi: 0003-2697(78)90586-9. PubMed PMID: 98070.

6. Mitchell PS, Parkin RK, Kroh EM, Fritz BR, Wyman SK, Pogosova-Agadjanyan EL, et al. Circulating microRNAs as stable blood-based markers for cancer detection. Proc Natl Acad Sci USA. 2008;105(30):10513-8. Epub 2008/07/30. doi: 10.1073/pnas.0804549105. PubMed PMID: 18663219; PubMed Central PMCID: PMC2492472.

7. Fichtlscherer S, De Rosa S, Fox H, Schwietz T, Fischer A, Liebetrau C, et al. Circulating microRNAs in patients with coronary artery disease. Circ Res. 2010;107(5):677-84. Epub 2010/07/03. doi: 10.1161/CIRCRESAHA.109.215566. PubMed PMID: 20595655.

8. Zampetaki A, Mayr M. Analytical challenges and technical limitations in assessing circulating miRNAs. Thromb Haemost. 2012;108(4):592-8. Epub 2012/05/26. doi: 10.1160/TH12-02-0097. PubMed PMID: 22627831.

9. Wagner J, Riwanto M, Besler C, Knau A, Fichtlscherer S, Roxe T, et al. Characterization of levels and cellular transfer of circulating lipoprotein-bound microRNAs. Arterioscler Thromb Vasc Biol. 2013;33(6):1392-400. Epub 2013/04/06. doi: 10.1161/ATVBAHA.112.300741. PubMed PMID: 23559634.

10. Schmittgen TD, Livak KJ. Analyzing real-time PCR data by the comparative C(T) method. Nat Protoc. 2008;3(6):1101-8. Epub 2008/06/13. doi: 10.3410/f.5500956.5467055. PubMed PMID: 18546601.

11. Kirschner MB, Edelman JJ, Kao SC, Vallely MP, van Zandwijk N, Reid G. The Impact of Hemolysis on Cell-Free microRNA Biomarkers. Front Genet. 2013;4:94. Epub 2013/06/08. doi: 10.3389/fgene.2013.00094. PubMed PMID: 23745127; PubMed Central PMCID: PMC3663194.

**Supporting Tables**

**Table A.** **Fold change values of circulating miRNA profiled in the pooled sera collected from CAD patients expressed relatively to Control group.**

|  | **Fold Change (versus Control group)** | | |
| --- | --- | --- | --- |
| **microRNA** | **SA** | **UA** | **MI** |
| **hsa-miR-486-5p** | 74.1307 | 31.5157 | 44.1702 |
| **hsa-miR-92a-3p** | 54.4930 | 26.3914 | 29.9399 |
| **hsa-miR-122-5p** | 15.7797 | 24.6925 | 29.6098 |
| hsa-miR-16-5p | 61.3503 | 14.9700 | 26.3732 |
| hsa-miR-25-3p | 37.8967 | 17.2557 | 19.5758 |
| hsa-miR-195-5p | 55.2920 | 14.5304 | 17.1365 |
| hsa-miR-221-3p | 22.0086 | 10.6960 | 13.6800 |
| hsa-miR-30a-5p | 23.4091 | 9.5732 | 12.2355 |
| hsa-miR-223-3p | 37.5307 | 15.8565 | 11.8105 |
| hsa-miR-93-5p | 23.8348 | 8.1851 | 11.7452 |
| hsa-miR-24-3p | 21.3180 | 9.2985 | 10.5050 |
| hsa-let-7d-5p | 10.6148 | 9.3567 | 9.3632 |
| hsa-miR-451a | 17.0063 | 6.1817 | 9.0130 |
| **hsa-miR-146a-5p** | 19.4136 | 7.7597 | 8.8520 |
| hsa-miR-30d-5p | 17.5208 | 6.3116 | 8.8214 |
| **hsa-miR-125a-5p** | 12.1257 | 8.0111 | 8.4855 |
| hsa-miR-30e-5p | 11.4082 | 4.8703 | 7.7866 |
| hsa-miR-423-3p | 7.8789 | 4.8266 | 7.4850 |
| hsa-let-7b-5p | 15.4229 | 7.9834 | 7.2904 |
| hsa-miR-23a-3p | 11.9671 | 6.1177 | 6.8069 |
| hsa-miR-22-3p | 3.8397 | 3.0398 | 6.5659 |
| hsa-miR-21-5p | 8.7604 | 6.0797 | 6.5387 |
| hsa-miR-140-5p | 12.3206 | 6.0293 | 6.4800 |
| hsa-miR-17-5p | 18.9746 | 5.8401 | 6.3908 |
| hsa-miR-15b-5p | 9.8970 | 4.5916 | 6.2463 |
| hsa-miR-29b-3p | 10.6442 | 2.2705 | 6.2333 |
| hsa-miR-125b-5p | 5.2964 | 3.1340 | 5.8038 |
| hsa-miR-424-5p | 7.5006 | 7.5947 | 5.6765 |
| hsa-miR-103a-3p | 6.1475 | 4.9760 | 5.5635 |
| hsa-miR-23b-3p | 5.5404 | 4.5504 | 5.5022 |
| hsa-miR-181b-5p | 5.2671 | 3.3173 | 5.4151 |
| hsa-miR-130a-3p | 9.8219 | 2.7856 | 5.4001 |
| hsa-miR-150-5p | 7.6000 | 3.6961 | 5.0246 |
| hsa-miR-222-3p | 3.6326 | 1.4419 | 4.9623 |
| hsa-miR-145-5p | 1.8661 | 2.0251 | 4.6978 |
| hsa-miR-107 | 4.5378 | 2.1600 | 4.6848 |
| hsa-miR-320a | 7.8953 | 5.2198 | 4.5473 |
| hsa-miR-100-5p | 9.8015 | 6.2636 | 4.4199 |
| hsa-miR-185-5p | 2.5829 | 1.2286 | 3.9231 |
| hsa-miR-30c-5p | 3.3612 | 2.9980 | 3.8745 |
| hsa-miR-27b-3p | 7.3513 | 3.9123 | 3.7503 |
| hsa-let-7a-5p | 6.4086 | 6.2290 | 3.6910 |
| hsa-miR-27a-3p | 6.7225 | 4.0139 | 3.5776 |
| hsa-miR-29c-3p | 4.4691 | 2.7530 | 3.2400 |
| hsa-miR-342-3p | 5.3480 | 4.4784 | 3.1296 |
| hsa-miR-144-3p | 5.2780 | 2.5456 | 3.0908 |
| hsa-let-7f-5p | 4.2604 | 4.5473 | 2.9506 |
| hsa-miR-378a-3p | 3.7581 | 1.2631 | 2.9201 |
| hsa-let-7c | 5.5174 | 4.6428 | 2.8520 |
| hsa-miR-29a-3p | 3.9231 | 2.4487 | 2.7207 |
| hsa-let-7e-5p | 5.5366 | 5.4717 | 2.7019 |
| hsa-miR-365b-3p | 10.8528 | 6.5750 | 2.5919 |
| hsa-miR-98-5p | 2.4743 | 0.7537 | 2.5333 |
| hsa-miR-26a-5p | 2.6099 | 2.8819 | 2.3295 |
| hsa-miR-126-3p | 3.9586 | 2.3867 | 2.3279 |
| hsa-miR-126-3p | 3.9586 | 2.3867 | 2.3279 |
| hsa-miR-181a-5p | 1.0981 | 3.0631 | 2.2439 |
| hsa-miR-26b-5p | 4.3379 | 4.3802 | 2.0677 |
| hsa-miR-210 | 6.8069 | 2.1258 | 2.0014 |
| hsa-miR-224-5p | 1.7938 | 2.0125 | 1.4980 |
| hsa-miR-143-3p | 4.9623 | 6.0002 | 1.3435 |
| hsa-miR-182-5p | 0.7961 | 0.8156 | 1.3050 |
| hsa-miR-10b-5p | 3.7503 | 2.9506 | 1.2834 |
| hsa-miR-99a-5p | 2.1795 | 2.5829 | 1.2466 |
| hsa-miR-142-3p | 0.9546 | 0.8202 | 1.2108 |
| hsa-miR-7-5p | 2.1555 | 0.9713 | 1.2000 |
| hsa-miR-494 | 1.9494 | 2.9938 | 1.1925 |
| hsa-miR-1 | 2.5071 | 1.1463 | 1.1925 |
| hsa-miR-133a | 0.7961 | 0.7537 | 1.1925 |
| hsa-miR-133b | 0.7961 | 0.7537 | 1.1925 |
| hsa-miR-149-5p | 0.7961 | 0.7537 | 1.1925 |
| hsa-miR-155-5p | 1.0253 | 0.7537 | 1.1925 |
| hsa-miR-183-5p | 0.7961 | 0.7537 | 1.1925 |
| hsa-miR-18b-5p | 0.7961 | 0.7537 | 1.1925 |
| hsa-miR-206 | 0.7961 | 0.7537 | 1.1925 |
| hsa-miR-208a | 0.7961 | 0.7537 | 1.1925 |
| hsa-miR-208b | 0.7961 | 0.7537 | 1.1925 |
| hsa-miR-214-3p | 1.0943 | 0.7537 | 1.1925 |
| hsa-miR-302a-3p | 0.7961 | 0.7537 | 1.1925 |
| hsa-miR-302b-3p | 0.7961 | 0.7537 | 1.1925 |
| hsa-miR-31-5p | 0.7977 | 0.7537 | 1.1925 |
| hsa-miR-499a-5p | 0.7961 | 0.7537 | 1.1925 |
| hsa-miR-124-3p | 0.7115 | 1.3416 | 1.1680 |
| hsa-miR-328 | 0.6708 | 1.0867 | 0.7433 |

Fold change values are the normalized miRNA expression (2-ΔCq) in the patient sample divided to the normalized miRNA expression (2-ΔCq) in Control sample. Normalized expression of a specific human miRNA is relative to that of exogenously added cel-miR-39 (ΔCq= Cqhsa-miRNA - Cqcel-miR-39). The miRNAs were ordered according to the fold changes measured in MI group (considered the control group for vulnerable CAD). The miRNAs selected for individual validation in the cohort’s CAD patients are shown in bold red. SA = patients with stable angina, UA = patients with unstable angina, MI = patients at 1 month after myocardial infarction.

**Table B.** **Distribution of the selected miRNAs in IDL and LDL fractions isolated by isopycnic density gradient ultracentrifugation from pooled sera of control subjects and CAD patients.**

| **Lipoprotein** | **miRNA** | **Control** | **SA** | **UA** | **MI** |
| --- | --- | --- | --- | --- | --- |
| **IDL** | **miR-122** | **ND** | **192.75**  ± 22.13 | **423.60**  ± 53.64 | **ND** |
|  | **miR-486** | **31.58**  ± 7.87 | **113.82**  ± 17.82 | **90.99**  ± 11.64 | **47.24**  ± 5.68 |
|  | **miR-92a** | **120.57**  ± 24.05 | **424.19**  ± 73.65 | **157.27**  ± 21.59 | **697.03**  ± 83.21 |
|  | **miR-125a** | **ND** | **19.41**  ± 12.91 | **ND** | **ND** |
|  | **miR-146a** | **30.22**  ± 7.64 | **65.03**  ± 7.82 | **ND** | **ND** |
|  | **miR-33a** | **34.74**  ± 8.25 | **73.86**  ± 4.59 | **62.81**  ± 19.69 | **141.15**  ± 21.17 |
| **LDL** | **miR-122** | **17.83**  ± 2.48 | **30.51**  ± 4.87 | **ND** | **ND** |
|  | **miR-486** | **11.17**  ± 18.24 | **11.47**  ± 3.27 | **ND** | **ND** |
|  | **miR-92a** | **13.67**  ± 5.45 | **172.72**  ± 44.16 | **ND** | **171.56**  ± 32.73 |
|  | **miR-125a** | **11.49**  ± 2.84 | **ND** | **ND** | **ND** |
|  | **miR-146a** | **ND** | 77.67  ± 11.04 | **ND** | **ND** |
|  | **miR-33a** | **78.16**  ± 12.78 | **94.99**  ± 24.74 | **173.01**  ± 21.84 | **156.99**  ± 23.54 |

Data are expressed as individual 2^-ΔCq^ values (relative to exogenously added cel-miR-39), multiplied with the same coefficient as for serum values (10^6^) and given as means ± standard deviation. The procedure was performed on 3 pools of sera from each group. SA = patients with stable angina, UA = patients with unstable angina, MI = patients at 1 month after myocardial infarction, ND = not detected.

**Supporting Figures’ Legends**

**Figure A. Hierarchical clustering plot (heatmap) of miRNA profiling in pooled sera from coronary artery disease (CAD) patients:** with stable angina (SA), unstable angina (UA), and patients at one month after myocardial infarction (MI), illustrated as coded colors for the fold change values expressed relative to Control sera. Red = maximum fold change (up-regulation), green = minimum fold change (down-regulation or no change).

**Supporting Figures**

**Figure A**

**
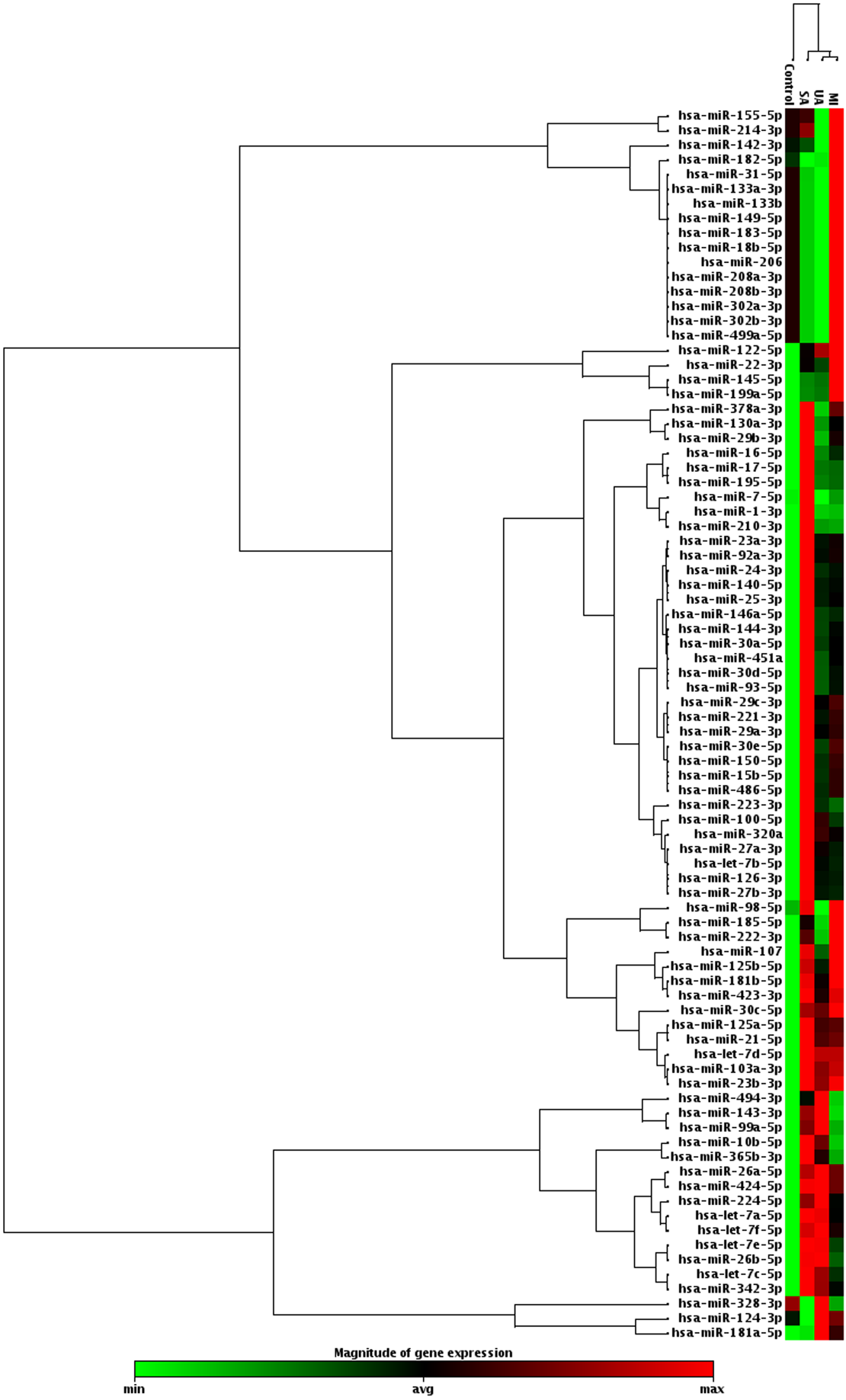
**
